# Arrhythmogenicity of fibro-fatty infiltrations

**DOI:** 10.1038/s41598-018-20450-w

**Published:** 2018-02-01

**Authors:** Tim De Coster, Piet Claus, Ivan V. Kazbanov, Peter Haemers, Rik Willems, Karin R. Sipido, Alexander V. Panfilov

**Affiliations:** 1 Department of Physics and Astronomy, Ghent University, Gent, 9000 Belgium; 2 Department of Cardiovascular Sciences, KU Leuven, Leuven, 3000 Belgium; 3 Moscow Institute of Physics and Technology, (State University), Dolgoprudny, Moscow Region, 141701 Russia; 4 Laboratory of Experimental Cardiology, Department of Cardiology, Heart Lung Centre Leiden, Leiden University Medical Center, Leiden, 2333ZA The Netherlands

## Abstract

The onset of cardiac arrhythmias depends on electrophysiological and structural properties of cardiac tissue. One of the most important changes leading to arrhythmias is characterised by the presence of a large number of non-excitable cells in the heart, of which the most well-known example is fibrosis. Recently, adipose tissue was put forward as another similar factor contributing to cardiac arrhythmias. Adipocytes infiltrate into cardiac tissue and produce in-excitable obstacles that interfere with myocardial conduction. However, adipose infiltrates have a different spatial texture than fibrosis. Over the course of time, adipose tissue also remodels into fibrotic tissue. In this paper we investigate the arrhythmogenic mechanisms resulting from the presence of adipose tissue in the heart using computer modelling. We use the TP06 model for human ventricular cells and study how the size and percentage of adipose infiltrates affects basic properties of wave propagation and the onset of arrhythmias under high frequency pacing in a 2D model for cardiac tissue. We show that although presence of adipose infiltrates can result in the onset of cardiac arrhythmias, its impact is less than that of fibrosis. We quantify this process and discuss how the remodelling of adipose infiltrates affects arrhythmia onset.

## Introduction

Abnormal excitation of the heart results in cardiac arrhythmias, which is a major problem in cardiac electrophysiology. Sudden cardiac death due to ventricular fibrillation remains the largest cause of death in the industrialized world^1^. Another cardiac arrhythmia, atrial fibrillation (AF) affects approximately 1.5% of the population^2^. AF is a progressive disease becoming more prevalent with increasing age and results in increased mortality, morbidity and impaired quality of life^3^. Therefore understanding the mechanisms and processes leading to the onset of cardiac arrhythmias is of great interest.

Over the years, several arrhythmogenic factors were identified. Among the most essential in them are the factors dealing with structural remodelling of cardiac tissue, resulting in the presence of in-excitable obstacles for wave propagation, such as fibrosis.

Fibrosis is characterized by the proliferation of non-excitable cardiac fibroblasts and an increase in the collagen fibres secreted by these fibroblasts. From an electrophysiological point of view, fibrosis is characterised by an increased number of in-excitable cells in the heart and occurs in many forms of heart disease^4–6^.

Recently, another similar factor was identified in the heart, namely adipose tissue^7^. Obesity was revealed to be an independent risk factor for the occurrence of AF or progression of AF severity in the absence of other risk factors like heart faillure, alcohol use or hypertension^8–10^. The risk of AF increases by 49% due to obesity in the general population, and the risk rises with an increased body mass index^11^.

Recent experiments have shown significant correlations between adipose infiltrations and arrhythmias^12,13^. Infiltrations of adipose tissue within the ventricular myocardium have been associated with arrhythmogenicity in arrhythmogenic right ventricular cardiomyopathy^14^ and more recently adipocyte infiltrations in the infarct border zone were associated with ventricular fibrillation^15^.

The specific mechanisms of arrhythmogenicity due to adipocyte infiltrations were not yet addressed in any study. From a general point of view, adipose tissue creates in-excitable regions inside the heart similar to fibrosis, and thus the mechanisms of arrhythmogenicity may be similar^16^. However, the fat cells (adipocytes) are much larger than fibroblasts and they are normally formed as compact infiltrates, which differs substantially from the texture of fibrotic tissue. Recently a new detailed study examined the relationship between fatty infiltrations in the atrial myocardium and the development of AF substrate^17^. Moreover, this study revealed a direct relation between fibrosis and adipose infiltrations. Progression of AF shows fibrotic remodelling of adipose infiltrates in the sub-epicardial layers of the atrial wall, associated with cellular inflammation. A transition from pure fatty to dense fibro-fatty infiltrations and smaller adipocytes was observed in the left atria of AF sheep. Therefore we see that adipose tissue is connected to fibrosis and it is important to understand how fibro-fatty remodelling affects arrhythmogenicity.

To answer these questions, one needs to study the onset of arrhythmias in cardiac tissue with controlled textures of adipose and fibrotic tissue, which is difficult to achieve in experiment. Therefore alternative approaches are of interest. One of them is computer modelling.

Computational models have been widely applied to study the mechanisms of arrhythmias in fibrotic tissue. The role of fibrosis in basic settings has been studied previously^18–20^. Its role in the initiation and maintenance of atrial fibrillation was demonstrated in an anatomically accurate model with realistic fibrosis distribution^21–23^. In relation to the onset of arrhythmias, the dependence of the re-entry probability on the texture of conducting clusters was reported as well^24^. A recent study shows detailed conditions of initiation of arrhythmias under high frequency pacing in fibrotic tissue and the role of fibrotic heterogeneity in it^25^.

In this paper, we perform an in-silico study on the onset of arrhythmias due to the presence of adipose tissue. We investigate the most basic effects and model adipose infiltrations as obstacles for wave propagation. We study how the size and density of adipose infiltrations affects the onset of cardiac arrhythmias and how it relates to arrhythmias occurring due to fibrosis. Finally we study a combined effect of fibrosis and fat infiltrations and discuss arrhythmogenicity due to fibro-fatty remodelling as observed in experiment^17^. Our simulations are based on the code developed by Kazbanov *et al*.^25^.

## Methods

### Mathematical model

Simulations were performed using the mono-domain equations for cardiac tissue:

1 $${C}_{m}\frac{dV}{dt}=-{I}_{ion}(V,\ldots )$$

*C*_*m*_ denotes the membrane capacitance, *V* the trans-membrane potential and *I*_*ion*_ is the sum of all ionic currents. These ionic currents depend on *V*, on the gating variables, and on the concentrations of intracellular calcium.

The spatial model can be described as:

2 $${C}_{m}\frac{d{V}_{ik}}{dt}=\sum _{\alpha ,\beta \in \{-\mathrm{1,}+\mathrm{1\}}}{\eta }_{ik}^{\alpha \beta }{g}_{gap}({V}_{i+\alpha ,k+\beta }-{V}_{ik})-{I}_{ion}({V}_{ik},\ldots )$$

where (*i*,*k*) is a position of a node occupied by a cardiomyocyte (because of the non-conducting character of fibroblasts and adipose tissue, computation of voltage is not needed in these nodes), *g*_*gap*_ is the conductance between two neighbouring myocytes. ${\eta }_{ik}^{\alpha \beta }$ is the connectivity tensor that describes the presence of electrical coupling between neighbouring myocytes and the absence of coupling between myocytes and fibrotic tissue, myocytes and adipose tissue, and fibrotic and adipose tissue:

3 $${\eta }_{ik}^{\alpha \beta }=\{\begin{array}{cc}1, & {\rm{i}}{\rm{f}}\,{\rm{t}}{\rm{h}}{\rm{e}}\,{\rm{n}}{\rm{o}}{\rm{d}}{\rm{e}}\,(i+\alpha ,k+\beta )\,{\rm{i}}{\rm{s}}\,{\rm{o}}{\rm{c}}{\rm{c}}{\rm{u}}{\rm{p}}{\rm{i}}{\rm{e}}{\rm{d}}\,{\rm{b}}{\rm{y}}\,{\rm{a}}\,{\rm{m}}{\rm{y}}{\rm{o}}{\rm{c}}{\rm{y}}{\rm{t}}{\rm{e}}\\ 0, & {\rm{o}}{\rm{t}}{\rm{h}}{\rm{e}}{\rm{r}}{\rm{w}}{\rm{i}}{\rm{s}}{\rm{e}}\end{array}$$

All studies were performed using the TP06 model of a ventricular cardiomyocyte. The second set of parameters from^26^ was chosen where no spontaneous break-up occurs due to dynamical instabilities to ensure the observed phenomena would be solely due to the presence of inexcitable tissue. Conductance *g*_*gap*_ was taken to be 103.6 *nS*, which results in a maximum velocity planar wave propagation, in the absence of fibrotic and adipose tissue, of $\mathrm{72\ }cm/s$ and $\mathrm{50\ }cm/s$ at stimulation frequencies of respectively 1 *Hz* and $4.1666\ldots Hz$ (periods of 1000 *ms* and $\mathrm{240\ }ms$).

### Fibrotic and adipose tissue distribution

The tissue structure consisted of a combination of myocytes, fibrotic tissue and adipose tissue. To construct the geometries, we used two parameters: percentage of adipose tissue *a* and percentage of fibrotic tissue *f*.

As a first step, the adipose tissue was superimposed on the healthy tissue. Circles of a specified radius were cut out of the healthy tissue where the center was randomly chosen in the tissue. This process happened under the condition that two circles did not overlap. This condition was based on the assumption that adipose tissue, upon making a connection, would merge into the shape of a larger circle. The algorithm to generate these geometries had an error margin of 1% with respect to the total tissue, since it was never possible to get to the exact percentage *a*. For each particular texture, we set up a maximal computational effort to generate it. If the placement of an additional infiltrate was longer than 2 CPU seconds, the further search was abandoned. This condition was never a problem for low percentages of non-conducting tissue. However, for percentages around 50% this condition resulted in low probability for generation of textures, thus in several graphs presented in the paper the percentage of non-conducting tissue does not go beyond 50%. This limitation can be overcome by setting up longer generation time. However, data points for extremely high non-conducting tissue percentages were not essential for the main conclusions of the paper.

In some simulations, we studied geometries containing adipose tissue with additional fibrotic tissue. In that case, a percentage of additional fibrotic tissue *f* was made non-conducting on a voxel based grid. In some simulations, we studied how the progression of fibrosis affects cardiac arrhythmias. For that, fibrotic tissue percentage was slowly increased by taking a previous geometry and adding to it a few extra percent of non-conducting tissue.

Both fibrotic and adipose tissue were assumed to be in-excitable and electrically disconnected from the myocytes, thus to be passive obstacles for wave propagation^16^. For our spatial model of a 2D cardiac tissue with interspersed fibrotic and adipose tissue, we used a rectangular grid with a size of 1024 × 1024 nodes, where each node can be occupied by either a cardiomyocyte or non-conductive tissue, consisting of fibrotic and adipose tissue. Thus, we assume that the area occupied by a cardiomyocyte is the same as the area occupied by fibrotic tissue, namely a square with an edge size of 75 *μm*. Since adipose tissue is larger in size, it was comprised of several connected non-conducting nodes.

**Figure 1:**
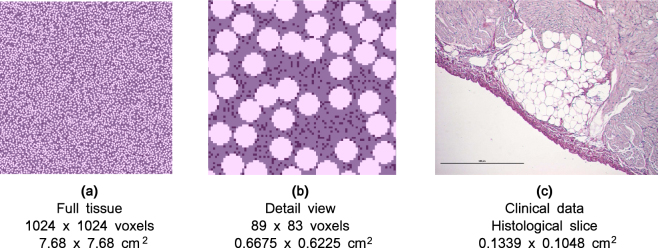
A comparison between simulated geometries and clinically observed ones. For clarity, a similar color scheme was used. Adipose tissue is the lightest color, myocytes the middle shade and fibrotic tissue has been given the darkest colour. In the simulation data (Fig. 1a and b), there was *a* = 40% adipose tissue and *f* = 9% fibrotic tissue for an adipose radius of 400 *μm*. The histological cut is from sheep data^17^ and has a scale bar which denotes 500 *μm*. Figure 1c shows one infiltrate which consists of individual adipocytes which can be seen as small circular shapes. Each circle in Fig. 1b accounts for the whole infiltrate and is not subdivided into individual adipocytes.

An example of a geometry with *a* = 40% adipose tissue and *f* = 9% fibrotic tissue is shown in Fig. 1. It can be seen that the texture is similar to an experimental cross-section. Percentage parameters were chosen to vary between geometries. The radii of the adipose infiltrations used in simulations (250 *μm* up to 700 *μm*) were based on experimental data which are in the range between 100 *μm* and 1000 *μm* approximately^17^. An example of a transmural section of one adipose infiltrate with a radius of approximately 400 *μm* is shown in Fig. 1c. For comparison with a circular geometry of an adipose infiltrate, fibrotic tissue was assumed to have a radius of 50 *μm*. Since it comprises of one voxel with an edge length of 75 *μm*, it can be regarded as a circular region with a radius of $\frac{75\sqrt{2}}{2}\mu m\approx 50\,\mu m$.

We performed 2D simulations which can be viewed as wave propagation in a subepicardial slice of cardiac tissue, i.e. parallel to the epicardial surface. Note though that the histological section shown in Fig. 1c is transmural, i.e. orthogonal to the epicardial surface.

To determine the percolation threshold, we initiated a wave at the left boundary of the tissue and studied if it reached the right boundary of the medium. The percolation threshold was taken as the smallest percentage of in-excitable tissue for which propagation failure was observed for all geometries. The percolation threshold is an important characteristic which was associated with onset of arrhythmias^24^.

### Implementation

The system of coupled ordinary differential equations (2) was solved by the forward Euler integration method, using a timestep of 0.005 *ms*. The equations for gating variables were integrated using the Rush-Larsen algorithm.

The numerical solver was implemented with the C and C++ programming languages, using the CUDA toolkit for performing the majority of computations on graphical processing units. Visualisation of results was done with the help of the Python programming language. Computations were performed with single precision and run on an Intel Core i7-3930K machine with three GeForce GTX TITAN Black graphics cards.

For each combination of non-conducting tissue, 10 textures were created. On most of the graphs the average values for these structures were plotted with the error bars denoting the standard deviation. When no error bar is visible, it is too small to be seen. Regions denoting ‘mixed region’ were also simulated extensively, but data points were omitted as the results extrapolate from the behaviour at the boundary.

For the detailed probabilities, every point was simulated with 20 different geometries with the spontaneous break-up protocol. The percentage of spontaneous break-up was plotted and fitted with Gaussian kernels.

To induce arrhythmias we used a pacing protocol consisting of 10 pulses at a period of 240 *ms*. If 1.68 *s* after the burst-pacing, activity was present in the preparation, the simulation was classified as causing arrhythmia onset (Supplementary Video S1).

To determine the boundary of the region of the onset of arrhythmia, we used the bisection algorithm. For every percentage of non-conducting tissue, between 10 and 20 simulations were executed on different geometries. When a percentage of at least 25% was found that caused spontaneous break-up at one end of the measurement, and lower than this percentage on the other, the search was deemed successful and the point was plotted together with its errorbar. Therefore, when the simulation was deemed unsuccessful, this either means the minimal 25% was not reached, or no geometry was possible to generate as was described above.

## Results

### Influence of adipose and fibrotic tissue on conduction velocity and spiral wave period

**Figure 2:**
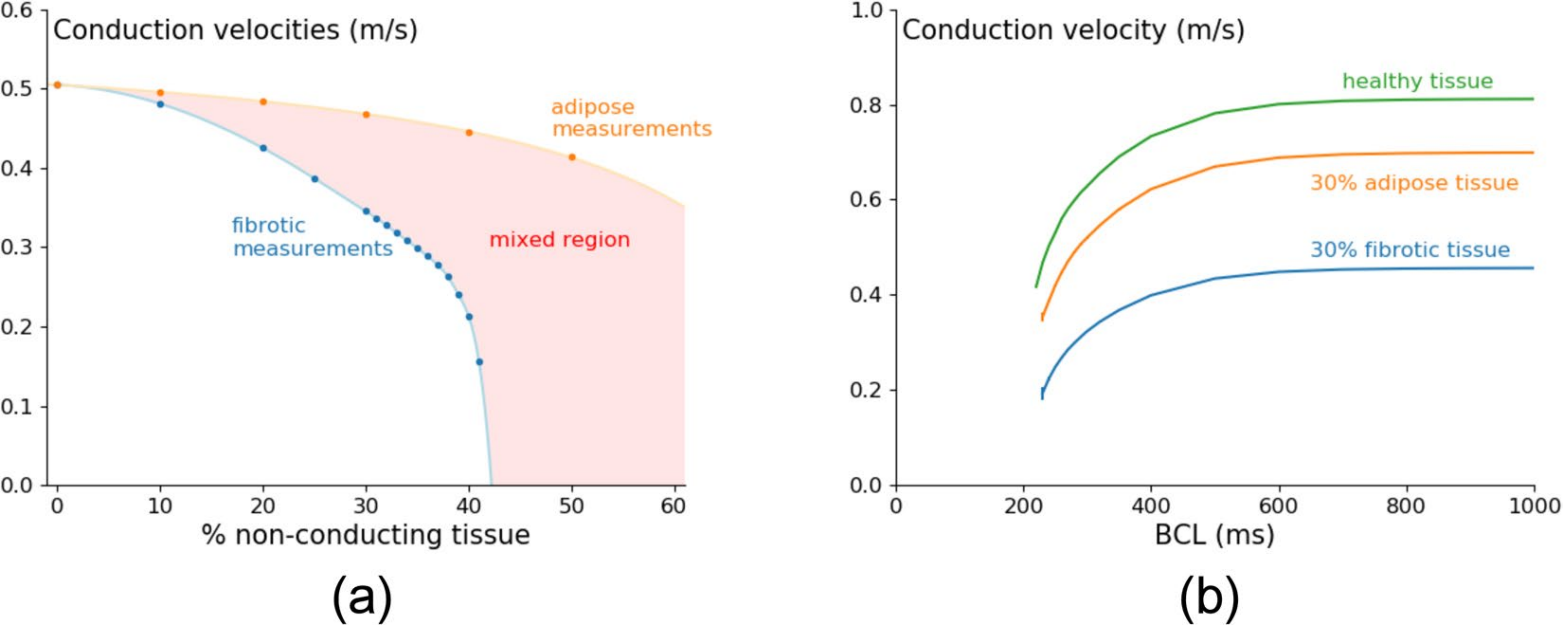
Conduction velocity at 240 *ms* periodical pacing for fibrotic and adipose tissue shows faster velocity decrease upon increase of fibrotic tissue in comparison with adipose tissue (**a**). The conduction velocity restitution curve of fibrotic and adipose tissue shows lower conduction velocities for fibrotic tissue than for adipose tissue during the whole stimulation range (**b**). Simulations were carried out in a tissue of area $76.8\times \mathrm{76.8\ }m{m}^{2}$, where the radius of fibrotic cells equals 50 *μm*, and of adipose infiltrates equals 400 *μm*. The conduction velocities in normal tissue are consistent with the velocities obtained in previous modelling studies^18,25^ and experimental recordings^38^. Note however that there is no data available for conduction velocities in the presence of adipose infiltrates.

We first study how the presence of adipose tissue affects conduction velocity (CV) for a pacing period of 240 *ms*, which is close to the typical period of arrhythmia in this model and compare it to the similar situation for fibrosis (Fig. 2).

In both cases, we see that velocity decreases with an increase of percentage of non-conducting tissue (Fig. 2a), and for all percentages the velocity with fibrotic tissue (F) is smaller than that for tissue with adipose tissue infiltrates (ATI). For ATI this dependency is also substantially less steep. For F the velocity dependency can be viewed first as gradual and then as rapid decrease close to 42% non-conducting tissue, which corresponds to the percentage where we observe wave propagation failure (the percolation threshold^24^). A CV restitution curve for 30% of non-conducting tissue can be seen in Fig. 2b. We see that the dependency of CV on frequency for ATI and F are similar and once again velocity with F is smaller than that for tissue with ATI.

**Figure 3:**
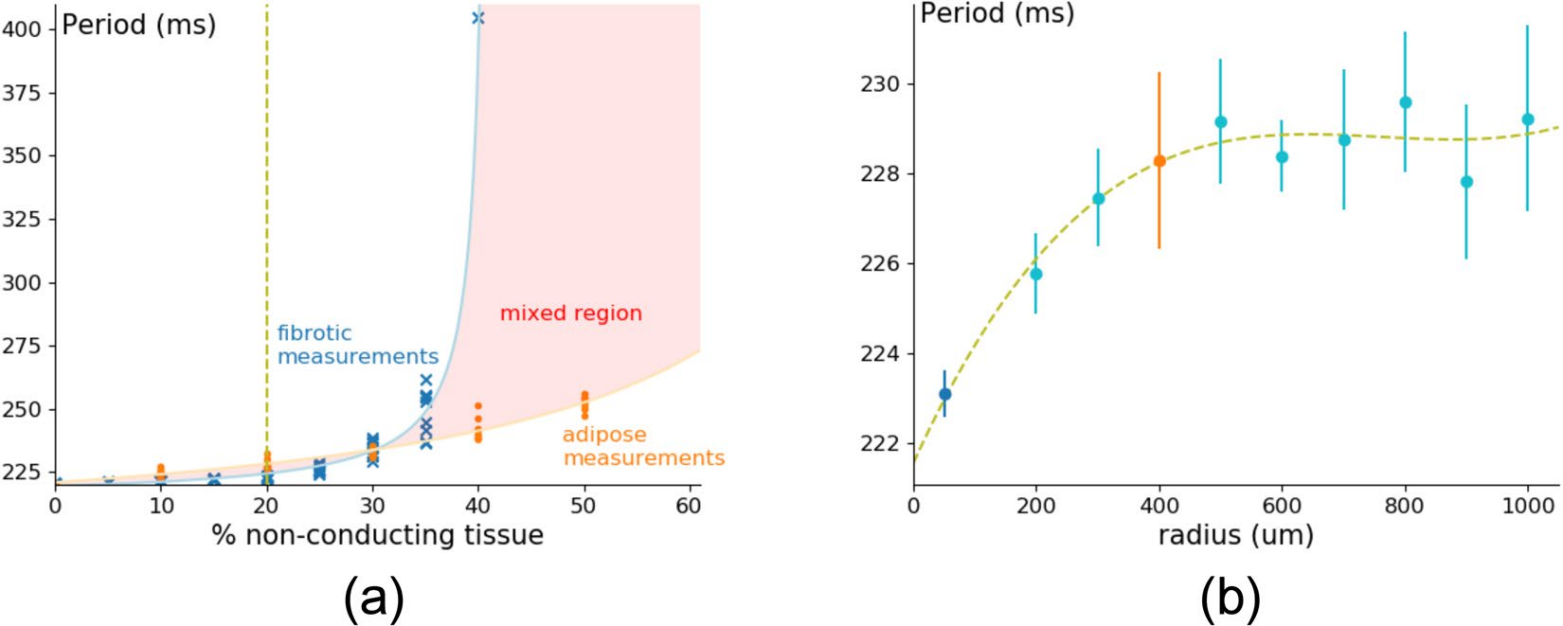
Period of spiral wave created by an S1S2 protocol for fibrotic and adipose textures with different percentages of non-conducting tissue (**a**) and with different sizes of adipose infiltrates in light blue (**b**). Simulations were carried out in a tissue of area $76.8\times \mathrm{76.8\ }m{m}^{2}$, where the radius of fibrotic cells equals 50 *μm*, and of adipose infiltrates equals 400 *μm* (**a**). The detail plot was made with the same tissue dimensions and for 20% of non-conducting tissue (**b**). The points for fibrotic and adipose tissue are plotted in their respective colour from Fig. 3a, dark blue (pure fibrotic tissue) and orange (pure adipose tissue).

Next we studied for the same preparations the effect of non-conducting tissue on the period of spiral wave rotation (Fig. 3).

An increase in the percentage of non-conducting tissue results in an increase of the period of the spiral wave (Fig. 3a). For small percentages of non-conducting tissue the periods for ATI and F preparations are close to each other. However, the period in F starts to grow faster and at 41.12% it becomes asymptotically large. This almost exactly corresponds to the percolation threshold. For ATI, a more gradual increase in the period is observed in that parameter range. In the range 0–30%, the period for ATI is slightly larger than for the case of F. Although this effect is small, it is counter-intuitive since the data for velocity (Fig. 2a) indicate a larger effect of F on wave propagation. To study this in more detail, the dependence of the period of spiral waves on the radius of the ATI was plotted in Fig. 3b for 20% of non-conducting tissue. An increase in the radius of the ATI indeed results in slight increase of the period of the spiral wave. The mechanism of this effect is not completely clear, as the typical radius of the core of a spiral wave in our cell model is around 10 *mm*, which is by far larger than the typical size of ATI. However, larger obstacles can still have micro influence on rotation of a spiral wave tip by helping to turn around them, which might result in a slight change in the period.

In Fig. 3a, the numerical results were fitted by the hyperbolic curve:

4 $$period=A+\frac{B}{percentage-C}$$

For F the parameters hold *A* = 214.39, *B* = − 213.48, *C* = 41.120, while for ATI with radius 400 *μm* the best fit values are *A* = 194.46, *B* = − 2434.1, *C* = 91.883. The parameters of the hyperbolic curve can have some meaning. In Eq. 4, the location of the vertical asymptote given by parameter *C* corresponds to the percolation threshold, as discussed above. Although for ATI we do not see a phase of rapid period increase, Eq. 4 predicts that it should occur for 91.9%. Interestingly enough, this almost exactly corresponds to so called perfect circular packing^27^, which gives a maximal density of 90.69%. However, we could not approach it in Fig. 3a as finding the configurations in which spiral wave activity could occur for large percentages of non-conducting tissue was beyond the reach of our algorithm, as described in the Methods section.

Overall, we can conclude that ATI has a smaller effect on wave velocity than fibrotic tissue. The effect on spiral wave period is similar. However, adipose tissue can still support spiral waves closer to the percolation threshold in fibrotic tissue (41.12%) and their period in that parametric range is much smaller than that in fibrotic tissue.

### Influence of adipose and fibrotic tissue on onset of arrhythmias

**Figure 4:**
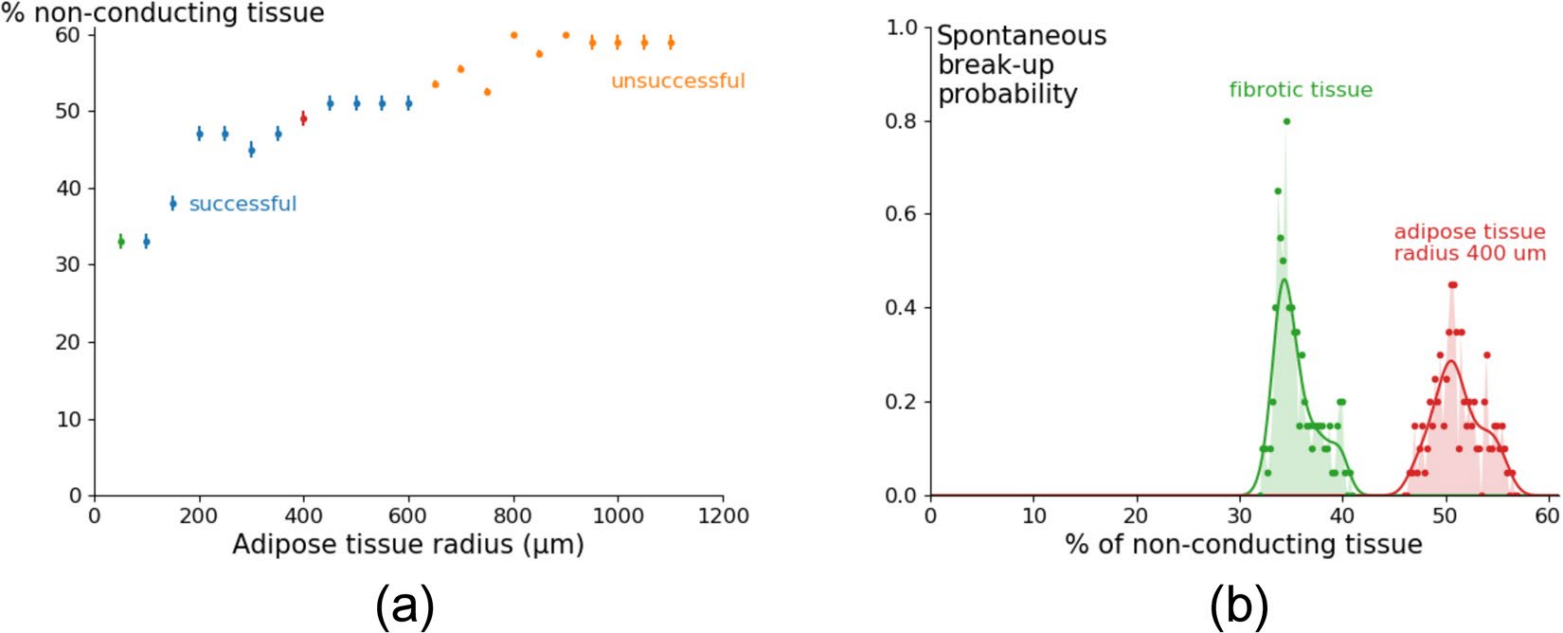
Generation of arrhythmia by high frequency pacing versus size of the fat infiltrates. The percentage of total in-excitable tissue is shown at which arrhythmia occurred and sustained for 1.68 *s* with a probability higher than 25% after 10 external stimuli with a period 240 *ms* (**a**). Detailed probability distribution for radius 50 *μm* and radius 400 *μm* (**b**). The peak of the distributions was found in Fig. 4a and was denoted by the same colour, green and red. Simulations were carried out in a tissue of area $76.8\times \mathrm{76.8\ }m{m}^{2}$, where the radius of adipose infiltrates equals 400 *μm*.

As one of the largest differences between ATI and F tissue is the size of the non-conducting regions, we studied how changing the size of ATI affects the onset of arrhythmias under a high frequency pacing protocol. The results of these simulations are shown in Fig. 4.

We found that for pure fibrotic tissue (radius 50 *μm*, the green point) the arrhythmia can be generated at 33% of non-conducting tissue. However, for adipose infiltrations of 400 *μm* (the dark red point) it occurs at 49%. Thus the onset of arrhythmia in the last case requires higher percentages and thus ATI can be considered as less arrhythmogenic. We see this tendency for all ATI radii: the percentage at which arrhythmia occurs increases monotonically with an increase in radius.

We also performed detailed analysis of the probability of break-up for 400 *μm* and compared it to fibrotic tissue (20 measurements every 0.25% for both cases). We see that the maximal probability of arrhythmia onset is larger for pure fibrotic tissue, although the interval at which arrhythmia occurs is wider for 400 *μm* radii (Fig. 4b). Due to this decreasing maximal probability, some searches were unsuccessful in Fig. 4a. Comparison of the total probability of arrhythmia onset (the area under the curve) shows that it is 1.132 times higher for fibrotic tissue than for adipose tissue. This indicates that adipose tissue is not only less arrhythmogenic in terms of percentage of non-conducting tissue but also in terms of total arrhythmia induction probability.

**Figure 5:**
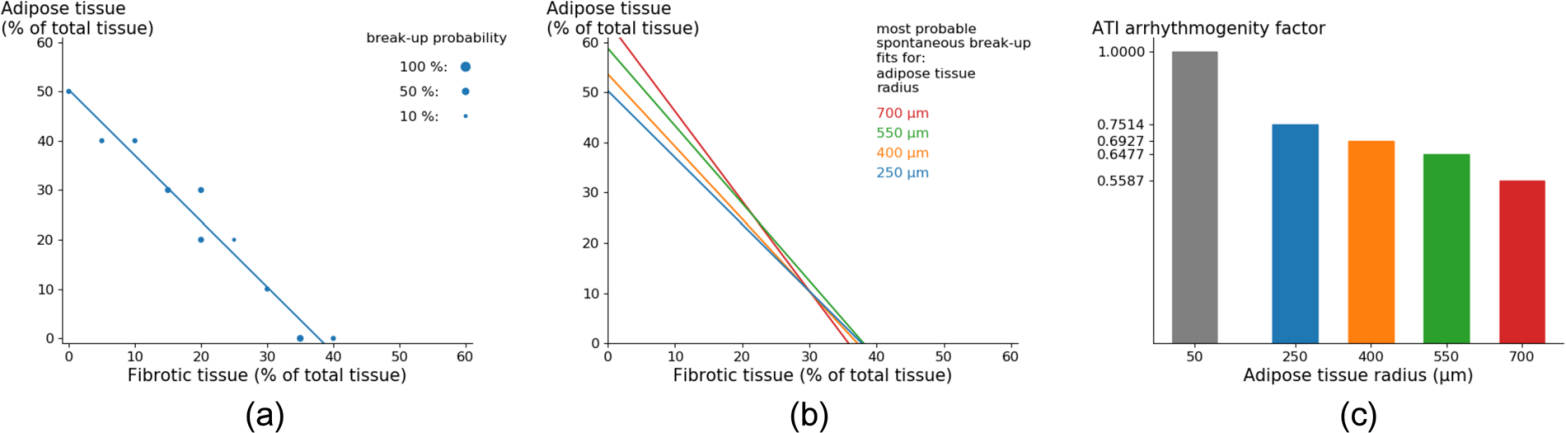
Textures of fat-fibrotic tissue corresponding to the most probable onset of arrhythmias for various radii of ATI and different percentage of ATI and F (Fig. 5b: The lines approximate the maximum of arrhythmia onset probability similar to that of Fig. 4b). The dependency can be fitted linearly. An example of such fit for radius 250 *μm* is presented (**a**). The comparison of arrhythmogenicity of ATI and F for various ATI radii represented by the arrhythmogeneity index (**c**).

As in most real situations infiltrations of adipose tissue occur in the presence of fibrosis, we studied how the combination of both adipose and fibrotic tissue affects the arrhythmia onset. To do so, tissue textures were generated which have a certain percentage of adipose tissue of given radius and a certain percentage of fibrotic tissue. Once again, induction of arrhythmias by high frequency stimulation was performed (Supplementary Video S1). The results are shown in Fig. 5.

A boundary of onset of arrhythmia for different radii of the ATI and for various percentages of ATI and F is presented (Fig. 5b). The dependency can be approximated by a linear fit (Fig. 5a). Since in the presence of only fibrotic tissue, the arrhythmias occur for the percentage of fibrosis around 35%, all curves in Fig. 5b cross the horizontal axis around this value. Since the percentage necessary for arrhythmia onset in the presence of only adipose tissue increases with the radius of ATI, the curves with larger radius cross the vertical axis at progressively larger values. Using this graph, it is possible to quantitatively compare the arrhythmogenicity of F and ATI by introducing the following index: from Fig. 5b we find that for radius 400 *μm*, for example, arrhythmia occurs for 10.00% F and 39.22% ATI. However, for 20.00% F it occurs for 24.79% ATI. Thus to keep the same arrhythmogenicity one can replace (39.22 − 24.79)% ATI by $\mathrm{(20.000}-\mathrm{10.000) \% }$ F and we can say that ATI is $\frac{20.000-10.000}{39.22-24.79}=0.6930$ times less arrhythmogenic than F. This index was computed for different radii (Fig. 5c). This factor decreases with increasing adipose radius, which not only indicates that ATI are less arrhythmogenic than F, but also assigns a quantity to it.

**Figure 6:**
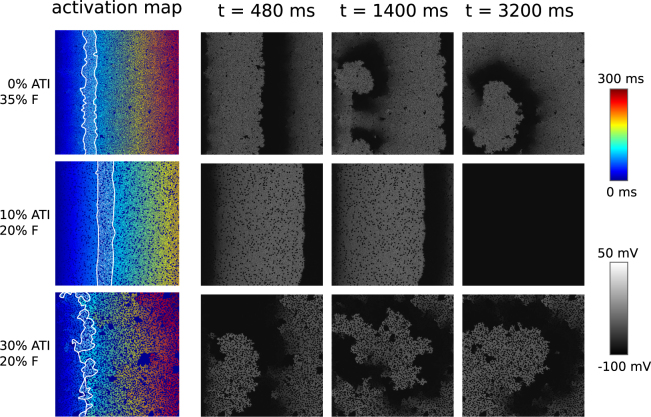
Initiation of arrhythmia using a burst pacing protocol for various ATI and F percentages. The left column shows activation maps for the first wave over a timespan of 300 *ms*. The white lines show the region activated during a time interval of 25 *ms* (from 75 *ms* till 100 *ms*). The gray scale pictures show the distribution of the transmembrane potential at given times *t*. Movies of all three simulations can be found in the supplementary information.

We also illustrate in Fig. 6 the process of arrhythmia formation under the aforementioned burst-pacing protocol for various ATI and F percentages. As a result of burst pacing we can have formation of multiple wavebreaks (the upper row) or a single wavebreak (the lower row). It has to be noted as well that for the tissue in the upper row it was more difficult to induce arrhythmia than for the tissue from the lower row: the wavebreaks appeared after the 5th and 2nd pacing stimulus in the upper and lower row respectively.

The ease of arrhythmia induction can be correlated with the complexity of the wavefront. It can be seen that in the upper tissue there are small deviations from a straight line in the wave-front, while the lowest one has large deviations from a straight line. The middle tissue has almost no deviations and we also did not observe wavebreak formation. Thus we can conclude that the more jagged the wavefront is, the more likely one is to observe spontaneous break-up.

The proximity of the white lines shows the local conduction velocity in the tissue. We observe that slower and spatially heterogeneous conduction correlates with the ease of arrhythmia induction.

Movies of all three processes shown can be found in the supplementary information.

### Transition of fat infiltrates towards fibrotic tissue

Let us apply our findings for the process of transition of fat infiltrates towards fibrotic tissue reported by Haemers *et al*.^17^. A shift from pure fatty to dense fibro-fatty infiltrations was observed in the left atria of AF sheep. During this process, we observe decreasing percentages of fat and increasing percentages of fibrosis. Let us consider what we expect in that situation based on our results.

**Figure 7:**
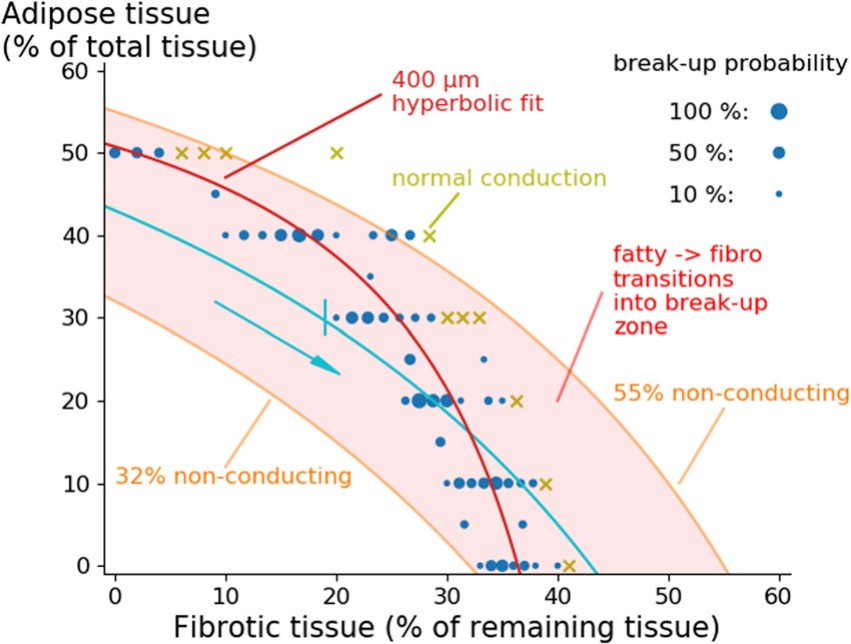
Schematic representation of arrhythmogenicity during fibro-fatty tissue remodelling. Further explanations can be found in the text.

In Fig. 7 we show detailed results of our simulations for an adipose radius of 400 *μm*. The blue circles of different radii represent the measured probabilities of arrhythmia onset. The best hyperbolic fit taking into account these onset percentages is shown in dark red. Note that the x-axis is now displaying fibrotic tissue with respect to the remaining tissue and not to the total tissue as in Fig. 5b. If the assumption is made that during remodelling of fat infiltrations to fibroblasts the total amount of non-conducting tissue (fat + fibrosis) remains constant, e.g. equal to 43% (the light blue line), then we expect the following changes during tissue remodelling:

If initially only adipose tissue is present, we start at the left of the graph and thus no arrhythmia is expected. During remodelling of fat to fibrotic tissue (following the blue curve), one would arrive to the place where arrhythmia can occur (near 30% ATI and 19% F). For further remodelling the arrhythmia probability only increases. However, if initially fat was 30%, a corresponding curve representing remodelling will not arrive to the arrhythmogenic region (not shown) and no arrhythmias would occur. Using such methodology we can effectively mark a vulnerable region, where the remodelling of adipose tissue towards fibrotic tissue can cause arrhythmias. This will occur when the curve crosses the region for break-up (Fig. 7). This region is shown by a light red colour and shows that for ATI with a radius of 400 *μm* we expect vulnerability for fat infiltrations from 32% to 55%.

## Discussion

In this paper we studied the effect of adipose tissue on arrhythmogenicity. Compared to fibrotic tissue, adipose tissue was found to be less arrhythmogenic in terms of percentage of non-conducting tissue needed to induce the arrhythmias and in terms of total breakup probability.

We modelled adipose infiltrates as non-conducting obstacles with no electrotonic connection to myocytes. We did this because so far no such connections were identified in experiment. Absence of such connections was also suggested in^16^. In addition, the resistivity of adipose cells is substantially larger than the one for myocytes^28^. Therefore, even if adipocyte-myocyte connections would exist, it will result in small currents which are unlikely to affect action potential generation in the myocytes.

Although we assume no electrotonic connection between adipocytes and myocytes, it has to be noted that adipocytes as well as fibroblasts produce biologically active molecules which can potentially affect the properties of myocytes^29–31^. This can result in additional pro- or anti-arrhythmic effects. For fibroblasts such effects have been studied in realistic three-dimensional human atria and it was shown that it results in the stabilisation of rotors^21,32,33^. It would be interesting to perform similar studies for adipocytes and fibro-fatty infiltrates.

In our simulations we always used a given constant percentage of in-excitable obstacles over the whole tissue. We expect however that similar results will hold for regional fat infiltrations. Thus predicted values for fat percentage can be viewed as local percentages of ATI as well.

### Limitations

We studied fat only as an in-excitable obstacle. However, adipose infiltrates produce a lot of biologically active molecules. They can therefore also affect the electrical properties of myocytes^34^. It would be interesting to construct a model based on these results with additional human measurements and use this in subsequent modelling research.

We considered 2D isotropic tissue with homogeneous ATI. In real situations, the ATI are 3D, heterogeneous and located at the boundary of the tissue. The 3D structure will definitely have an important effect on the process of arrhythmia generation and needs to be further investigated. Based on the results of our paper we can assume the following. Since adipose infiltrates protrude from the epicardial surface, the largest percentage of adipose tissue will be in the epicardial layer of the heart and will decrease towards the midmyocardium. Thus we expect that arrhythmia will be initiated at some layer with an obstacle distribution where the wavebreaks are more likely to occur as presented here, which will be most probably close to the epicardial surface. Subsequently wavebreaks will evolve in 3D tissue and may result in complex 3D patterns of excitation. A first step to study 3D phenomena could be to simulate these conditions in 3D-slabs and then in anatomically accurate models of ventricles and atria.

All our studies were performed using a human ventricular cell model. However, the properties of adipose infiltrates were taken from sheep atrial data. In ventricular disease, adipose tissue infiltrates^35^ have been studied in the context of ARVD^36,37^ and peri-infarct remodelling^15^. These data show that adipose infiltrations occur from outside inwards in a fashion that was also observed in the sheep atria. Unfortunately these papers do not provide a size distribution of the infiltrates. It can be seen however that the topology of the infiltrations in sheep and human data is similar. Since we performed our study in a wide range of adipose radii, our results should cover the possible sizes of the infiltrates in human ventricular tissue. However, it would be interesting to perform studies based on quantitative human data, when they become available.

When predicting arrhythmogenicity during remodelling, the total amount of non-conducting tissue (ATI + F) was considered to be constant. There are no measurements that confirm or deny this assumption. The results presented by Haemers *et al*.^17^ only show that the adipose tissue percentage decreases with increased fibrotic remodelling, while myocardial fibrosis (measured with respect to the healthy tissue) increases.

## Electronic supplementary material

Break-up mechanism for different tissue structures
